# How to promote female medical leaders? Insights and solutions from the JACRA Chief Resident Meeting

**DOI:** 10.1002/jgf2.70022

**Published:** 2025-04-17

**Authors:** Masumi Ogushi, Kazuya Nagasaki, Toshinori Nishizawa, Yuko Murashima, Kosuke Tanaka, Takuma Hata, Masayuki Nogi

**Affiliations:** ^1^ General Medicine Research Beth Israel Deaconess Medical Center Brookline Massachusetts USA; ^2^ Department of Internal Medicine Mito Kyodo General Hospital, University of Tsukuba Ibaraki Japan; ^3^ Department of General Internal Medicine St. Luke's International Hospital Tokyo Japan; ^4^ Department of Public Health Institute of Science Tokyo Tokyo Japan; ^5^ Department of Gastroenterology St. Luke International Hospital Tokyo Japan; ^6^ Department of Real World Data Research and Development Graduate School of Medicine, Kyoto University Kyoto Japan; ^7^ Department of General Internal Medicine Hakujyuji General Hospital Ibaraki Japan; ^8^ Division of Hospital Medicine Queen's Medical Center Honolulu Hawaii USA; ^9^ Department of General Internal Medicine Kameda Medical Center Chiba Japan

To the Editor,

Reports from the United States underscore the vital role of chief residents in clinical practice and medical education, serving as leaders for their fellow residents.^1^ While early leadership experience offers numerous benefits, a significant challenge persists in Japan: leadership positions remain predominantly held by men. Beyond leadership roles, Japan grapples with a pervasive gender gap within its medical field. For instance, the proportion of female physicians in Japan is the lowest among OECD countries, standing at just 23.6%.^2^ This leadership disparity extends across hospital executive boards, professional societies, and academic institutions, limiting diversity at decision‐making levels.

At the JACRA (Japan Chief Resident Association) Chief Resident Meeting in March 2024, about 40 current and incoming chief residents convened to discuss two key questions: “Why are there so few female leaders?” and “What actions are necessary to foster gender‐diverse leadership?” To encourage candid feedback, an anonymous online polling system was utilized. Table 1 presents a categorization of audience responses. Results highlighted key challenges such as work‐life balance struggles and inadequate leadership training. Many participants emphasized the unequal distribution of clinical and administrative workloads among female leaders. This letter presents insights from the discussion and potential solutions to advance gender equity.

**TABLE 1:** Categorized ideas from audiences.

Categories	Why few female leaders?	What is needed for diverse leaders?
Gender gap and bias	Imposter phenomenon	Increase awareness of imposter phenomenon
	Society's hesitation to change current leadership landscape	Change the perception that predominantly male leaders is not normal
	Perception that women tend to be humble and not a standout	Select a leader through endorsements
	Imbalance of male to female physician ratio, contributing to leadership gender gap	Increasing the number of female physicians
	The authorities in medicine field are mostly men	Support from the organization and colleagues
	From a young age, women are not culturally expected to take on the leader role	Increase the presence of female young leaders in the society
The impact of life events	Career is interrupted by childbirth, taking care of house and family	Create a supportive childcare environment
		Reducing the burden on single physicians who cover for other physicians with childcare responsibilities
	Men rarely take parental leave	Promote parental leave regardless of gender
	Society expects women to handle childcare	Outsource housework and childcare
		Increase extended family's support
Leadership support system	Lack of leadership education	Increase leadership education opportunities for women
	Many meetings outside of working hours	Hold meetings within working hours
	Leaders lack protected time for administrative tasks	Promote task sharing of leadership and clinical duties
		Reduce clinical duties for leaders
		Increase institutional support for compensation and protected time
		Establish quota system for leaders
	Differences in endurance and resilience to long work hours	Foster a culture that accepts diverse and flexible workstyles

Participants noted that traits such as modesty and humility, often associated with women, contribute to their hesitation in assuming leadership roles. The “imposter phenomenon,” characterized by a tendency to underestimate one's abilities, was also frequently mentioned.^3^ These observations underscore deep‐rooted biases affecting leadership perceptions and career advancement.

The physical demands on women and their career impact were recurring themes. Mid‐career breaks due to childbirth and childcare responsibilities pose major obstacles, affecting promotion opportunities. Matsui et al. found that female medical students prioritize family‐career balance when making career choices, whereas male students focus on work initially and adjust later.^4^

Systemic barriers also hinder women's advancement. Female leaders often face excessive clinical and administrative workloads, compounded by insufficient institutional support. Limited networking opportunities and lack of visibility within professional circles further exacerbate these challenges, making career progression more difficult.

Addressing these issues requires a comprehensive, multi‐pronged approach. Institutions must take proactive steps to increase female representation in leadership. Establishing mentorship programs, particularly group mentorship, can foster confidence, skill development, and career progression. Systemic reforms such as promoting shared family responsibilities, expanding parental leave for men, and preventing overwork are essential. Restructuring leadership roles by implementing collaborative leadership models and alleviating administrative burdens would also allow leaders to focus on core responsibilities and professional growth.

Research demonstrates the benefits of gender‐diverse leadership in healthcare. Naciti et al. (2022) found that hospitals with more female leaders achieved better financial performance, enhanced efficiency, and improved decision‐making.^5^ These findings emphasize the need to foster gender diversity to enhance healthcare quality and efficiency.

JACRA remains committed to supporting these efforts through targeted leadership education and training initiatives. By tackling systemic barriers, enhancing mentorship, and fostering inclusivity, we aim to empower women to take on leadership roles, contribute their expertise, and drive meaningful change in the medical profession.

## NOTATION OF PRIOR ABSTRACT PUBLICATION/PRESENTATION

This work has not been presented.

## AUTHOR CONTRIBUTIONS

**Masumi Ogushi:** Writing – original draft; writing – review and editing; resources; project administration; visualization; conceptualization; methodology; investigation. **Kazuya Nagasaki:** Supervision; writing – review and editing; project administration. **Toshinori Nishizawa:** Conceptualization; investigation; supervision; project administration; writing – review and editing; methodology. **Yuko Murashima:** Investigation; conceptualization. **Kosuke Tanaka:** Conceptualization; investigation. **Takuma Hata:** Conceptualization; investigation; methodology; supervision; project administration. **Masayuki Nogi:** Writing – review and editing; resources; project administration.

## FUNDING INFORMATION

This letter did not receive external or internal financial support.

## CONFLICT OF INTEREST STATEMENT

The authors have no conflicts of interest to declare.

## CONSENT

We confirm that agreement for publication was obtained from the subject of the Figure.
